# A Cross-Sectional Study of Heat Wave-Related Knowledge, Attitude, and Practice among the Public in the Licheng District of Jinan City, China

**DOI:** 10.3390/ijerph13070648

**Published:** 2016-06-29

**Authors:** Jing Li, Xin Xu, Guoyong Ding, Yun Zhao, Ruixia Zhao, Fuzhong Xue, Jing Li, Jinghong Gao, Jun Yang, Baofa Jiang, Qiyong Liu

**Affiliations:** 1 Department of Epidemiology and Health Statistics, School of Public Health, Shandong University, Jinan 250012, Shandong, China; lijingsddx@126.com; 2 State Key Laboratory of Infectious Disease Prevention and Control, Collaborative Innovation Center for Diagnosis and Treatment of Infectious Diseases, National Institute for Communicable Disease Control and Prevention, Chinese Center for Disease Control and Prevention, Beijing 102206, China; lijing986169323@sina.cn (J.L.); gaojinghong2007@126.com (J.G.); smart_yjun@163.com (J.Y.); 3 Shandong University Climate Change and Health Center, Jinan 250012, Shandong, China; 4 Department of Dentistry, Affiliated Hospital, Weifang Medical University, Weifang 261000, Shandong, China; sl_1@163.com; 5 School of Public Health, Taishan Medical College, Taian 271000, Shandong, China; dgy-153@163.com; 6 Licheng District Center for Disease Control and Prevention, Jinan 250012, Shandong, China; zhaoyunepi@163.com (Y.Z.); zh.rx@163.com (R.Z.); 7 Department of Biostatistics, School of Public Health, Shandong University, Jinan 250012, Shandong, China; xuefzh@sdu.edu.cn

**Keywords:** knowledge, attitude, practice, heat waves, China, cross-sectional study

## Abstract

Knowledge, attitude, and practice (KAP) are three key components for reducing the adverse health impacts of heat waves. However, research in eastern China regarding this is scarce. The present study aimed to evaluate the heat wave-related KAP of a population in Licheng in northeast China. This cross-sectional study included 2241 participants. Data regarding demographic characteristics, KAP, and heat illnesses were collected using a structured questionnaire. Univariate analysis and unconditional logistic regression models were used to analyze the data. Most residents had high KAP scores, with a mean score of 12.23 (standard deviation = 2.23) on a 17-point scale. Urban women and participants aged 35–44 years had relatively high total scores, and those with high education levels had the highest total score. There was an increased risk of heat-related illness among those with knowledge scores of 3–5 on an 8-point scale with mean score of 5.40 (standard deviation = 1.45). Having a positive attitude toward sunstroke prevention and engaging in more preventive practices to avoid heat exposure had a protective interaction effect on reducing the prevalence of heat-related illnesses. Although the KAP scores were relatively high, knowledge and practice were lacking to some extent. Therefore, governments should further develop risk-awareness strategies that increase awareness and knowledge regarding the adverse health impact of heat and help in planning response strategies to improve the ability of individuals to cope with heat waves.

## 1. Introduction

The Fifth Report of the Intergovernmental Panel on Climate Change stated that the average global surface temperature increased linearly by 0.85 °C from 1880 to 2012 [1]. Therefore, it is highly possible that the frequency and duration of heat waves will increase in the future. Heat-related diseases may affect the health of billions of people at risk, and will likely affect most populations in the coming decades [2]. The adverse effects of elevated temperature on mortality have been widely reported. For example, in 2012, heat caused more fatalities than any other severe weather event worldwide [3], and a study from USA reported that 8015 deaths were directly caused by heat waves from 1979 to 1999 [4]. Other studies have reported that the 1995 heat wave in Chicago was alone responsible for over 800 deaths [5], and that the heat wave in 2003 across Europe was associated with 22,000 to 35,000 additional deaths [6]. Heat waves have been reported to introduce health risks in some areas in China. The 2003 heat wave in Shanghai was believed to be associated with high respiratory and cardiovascular mortality rates [7]. Furthermore, extremely high temperature in Guangzhou was linked with an increase in the risk of death [8]. Elderly, women, and individuals with pre-existing diseases are considered to be very vulnerable to heat-related illnesses in China [9,10]. Therefore, more attention should be paid to the health effects of heat and heat waves.

A “Knowledge, Attitude, and Practice” (KAP) study is performed in a specific population to collect data on what the population knows, believes, and does regarding a certain topic. The public’s attitude to risks, adaptation practices, and knowledge of heat waves are three of the most important factors for reducing the health impact of heat waves [11,12]. KAP studies on heat waves have been mostly performed in developed countries [13,14,15], and relatively few studies have been performed in developing countries, especially in China. Yet, according to the Global Climate Risk Index 2014, China is vulnerable to extreme weather events [16]. Therefore, it is important to understand the Chinese public’s KAP in order to reduce the negative effects of heat waves.

The public’s KAP can be influenced by several aspects, such as climate type, economic level, and public characteristics. Although KAP data are sensitive, the findings can be considered as local indicators that are only representative in a defined and limited area. KAP studies on heat waves have been performed in the south and west regions of China [12,17]. However, KAP studies on heat waves have not been performed in the east region. Heat waves have become increasingly common in Jinan City. From 1951 to 2005, 763 days had high temperatures (≥35 °C) [18]. According to the 2011 Statistical Yearbook of Jinan City, the maximum daily temperature in summer has continuously increased in recent years, and the maximum temperature reached 41.4 °C in the summer of 2009 [19]. Owing to the continuously high temperature in this region, it is necessary to perform a public survey to evaluate the health impact of heat waves so that the number of deaths from heat waves can be reduced. The results could contribute to the understanding of the public‘s KAP with regard to heat waves, which would in turn provide data to local health authorities and communities for future policy-making and implementation in terms of coping with heat waves and the corresponding adverse effects.

The present study aimed to obtain data in order to better understand the heat wave-related KAP of a population in Licheng District of Jinan City in the northeast region of China. We selected Licheng District as the study area because its demographic characteristics, climate type, and population lifestyle are representative of Jinan City [19]. The results will be further evaluated so that they can be used to develop policies for coping with heat waves.

## 2. Methods

### 2.1. Study Area and Definition of Heat Waves

**Figure 1:**
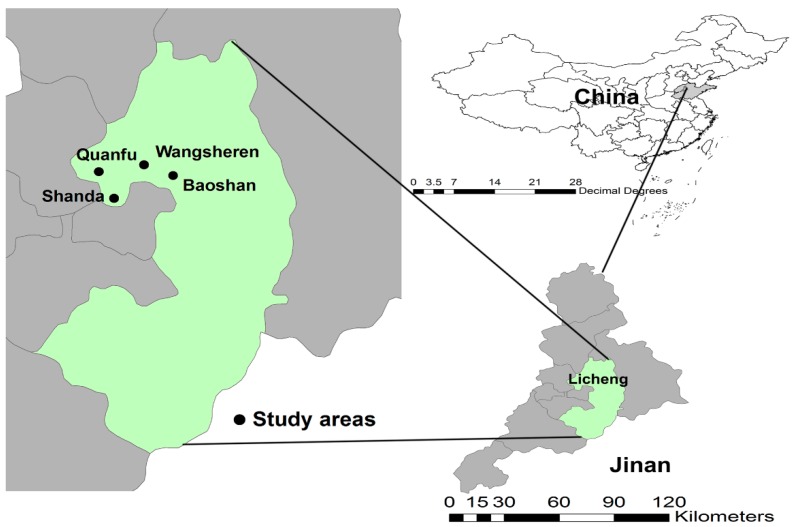
The four sample streets in Licheng District, China.

Licheng District is located in the southeast area of Jinan City (geographical coordinates, 36°40′ N and 117°03′ E, Figure 1). The area of the district is 1298.57 km^2^, with a mean elevation of 41 m and a total population of 1.12 million. At the end of 2010, the sex ratio was 99.6:100 (male:female). The climate of the district is characterized as sub-humid and warm, with obvious continental monsoon weather and four distinct seasons. The annual mean temperature, precipitation, and sunshine hours are 13.8 °C, 685 mm, and 5.1 h, respectively. Licheng District has 15 streets, with 47 community resident committees and 655 administrative villages. Individuals living in the communities mainly work in industries and related services, and those living in the villages mainly grow cash crops. Jinan City is called one of the four “ovens”. According to the Chinese Meteorological Administration, a “heat day” is defined as a day when the maximum temperature exceeds 35 °C, and a heat wave is a period of at least three consecutive “heat days” [7]. This definition was adopted in the present study.

### 2.2. Study Participants

The multistage sampling method was utilized to select a representative sample in Licheng District. First, the district was divided into an urban area and a rural area according to the density of population and vegetation cover. Then, two streets were selected non-randomly from each area that had similar demographic characteristics and economic status. The four selected streets were Shanda, Quanfu, Baoshan, and Wangsheren (Figure 1). Shanda and Quanfu were from the urban area, and Baoshan and Wangsheren were from the rural area. We further selected 600 households from each street randomly. Finally, individuals who were over 14 years old and had been living in the selected areas for at least 6 months were considered eligible for inclusion in the present study. One eligible participant was selected from each household using the Kish grid method.

### 2.3. Data Collection

A questionnaire was drafted after a review of the literature on heat waves and climate change [13,15,17,20,21]. Experts were consulted during the development of each question and its response. The following five sections were present in the questionnaire: (1) section “A” (questions on demographic information, such as gender, age, education, and marital status); (2) section “B” (eight questions on knowledge about heat waves); (3) section “C” (one attitude-related question, i.e., whether measures were taken to prevent heatstroke if a high temperature warning had been provided); (4) section “D” (four questions on practices for preventing heat waves and previous experiences of heat-related diseases); and (5) section “E” (questions on economic and social factors, such as dwelling environment, cooling system, and neighborhood relationships).

From 12 to 18 July 2014, each participant was interviewed at home by a well-trained investigator using the structured questionnaire. Various actions were taken to ensure the quality of the questionnaire. First, all interviewers received systematic training for several days before the survey. Training provided the interviewers with an opportunity to become familiar with the survey objectives, content, and plan for implementation in the study. The training mainly included the following areas: survey purpose; roles and responsibilities; content and use of the questionnaires; survey forms and materials; appropriate interviewing techniques, including listening skills and probing techniques; and final pre-testing of the questionnaire. A small-scale pilot study was performed to assess the validity and reliability of the questionnaire. Moreover, the questionnaire and the research methods were thoroughly discussed within the research team. Feedback was carefully reviewed, and appropriate modifications were made to the questionnaire. Furthermore, four senior researchers were always present during questionnaire collection to monitor the process and examine the quality of the data collected.

### 2.4. Statistical Analysis

There were eight, one, and four questions regarding knowledge (K), attitude (A), and practice (P), respectively. For the K and P sections, the scores were assessed by assigning 1 point to each response of “Yes” and 0 point to response of “No.” For the A section, a 5-point scale was used as the marking scheme, corresponding to the five options of the questions (the options from “not at all” to “very much” were scored from 1 to 5). The score ranges for the K, A, and P sections were 0–8, 1–5, and 0–4, respectively. The total KAP score was the sum of the scores of the three components, and the score range was 1 to 17. 

To identify the occurrence of a heat-related illness, which was the dependent variable in our study, every participant was asked if they felt discomfort due to high temperature and visited a doctor; if they showed self-medication behavior to cope with the heat; if they had been diagnosed with heatstroke by a clinical doctor; or if they had any symptoms of heatstroke during the heat wave period in a particular year. Those who responded “yes” to any of the above questions were considered to have experienced heat-related illnesses in this study.

Mean and standard deviation (SD) values were calculated for continuous variables, such as age and KAP score. Categorical variables were computed as a percentage of subjects with the perspective attribute. First, univariate analysis of variance was used to test the association of each demographic characteristic with the K, A, and P scores and the total score. Then, Pearson correlation analysis was performed to clarify the correlations among the K, A, and P scores. In the next stage, participants were divided into three groups according to the K score (<3, 3–5, and >5), two groups according to the A score (<3 and ≥3), and two groups according to the P score (<3 and ≥3). Unconditional logistic regression models (I and II) were used to assess the associations of the K, A, and P scores with heat-related illnesses. Model I assessed the main effects of the K, A, and P scores with heat-related illnesses, without confounding variables, whereas model II assessed the main effects of the K, A, and P scores with heat-related illnesses after adjusting for confounding variables. A product term (K × A, A × P, and K × P) of KAP was further added into the logistic models to test their interaction effects on heat illnesses. The new models were model III (without confounding variables) and model IV (with confounding variables). The interaction effects were analyzed in any two groups of K, A, and P using the low score group as a control. Adjusted confounding variables included sex, age, education level, marital status, occupation, monthly income, and Hukou with a registered permanent residence. All statistical analyses were performed using SPSS 21.0 (SPSS Inc., Chicago, IL, USA), and a *p*-value < 0.05 was considered statistically significant.

## 3. Ethical Statement

Ethical approval was obtained from the Ethics Committee of the Chinese Center for Disease Control and Prevention (No. 201214). Written informed consent was obtained from all the participants prior to the survey. All data obtained were anonymous. 

## 4. Results 

### 4.1. Demographic Information

In the baseline survey, the number of people approached was 2400 and the response rate was 93.4% (2241/2400). The characteristics of the 2241 survey participants are presented in Table 1. Among the 2241 participants, 45.6% were male and 61.5% were from urban areas. The mean age of the participants was 43.5 years (15–91 years, SD = 16.55). Most participants (82.3%) were married. The education level of the participants varied. With respect to monthly income, 33.8% of the participants had a monthly income of 2000–3000 Yuan (US$324) and 1.8% had a monthly income of more than 10,000 Yuan (US$1620).

### 4.2. Responses to Questions on Heat Wave-Related Knowledge, Attitude, and Practice

Table 2 shows the responses to the questions on heat wave-related KAP among the survey participants. Half of the questions in the K section received accurate responses from over 80% of the participants. Most of the participants knew that fans and sprinklers in open grounds could help keep cool (82.3%), and were aware of some of the common symptoms of heat stroke, including fever and fatigue (82.9%). However, less than half of the participants did not know that some medicines could increase the risk of heatstroke (42.2%). Over two-third of the participants considered that the greenhouse effect was mainly caused by the depletion of the ozone layer. 

The question in the A section had five responses. The ratio of participants who answered positively (selected “very much” or “much” for prevention measures) to the question was 66.7%. Less than 1% of the participants answered “not at all.”

In the P section, 90.2% of the participants stated that it was necessary to pay more attention to special populations. Additionally, over 80% of the participants were aware that it was necessary to perform activities at cooler times and to take appropriate and adequate preventive measures when going out. However, 56.1% of the participants reported drinking water only when thirsty.

### 4.3. Mean Scores of Heat Wave-Related Knowledge, Attitude, and Practice

**Figure 2:**
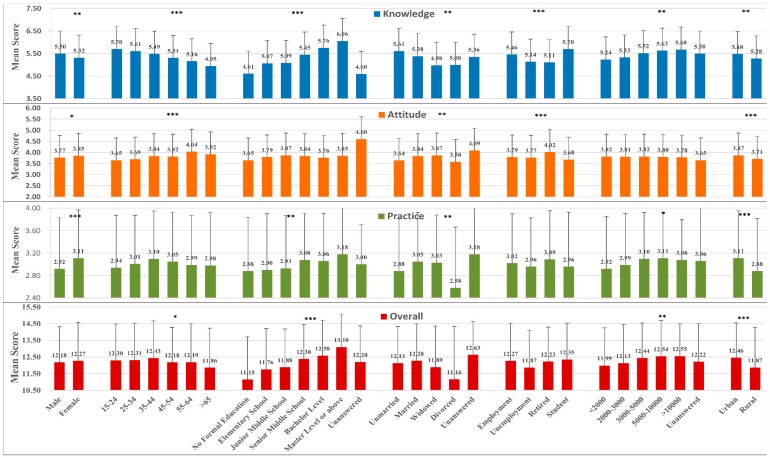
Mean KAP scores according to the demographic characteristics among the participants in Licheng District. * *p* < 0.05, ** *p* < 0.01, *** *p* < 0.001.

The detailed mean K, A, and P scores according to demographic characteristics are listed in Figure 2. The mean K score of all participants was 5.40 (SD = 1.45). There were noticeable differences in the mean K score among participants with respect to all demographic factors (*p* < 0.05). The score was higher in male participants than in female participants (5.50, SD = 1.39 vs. 5.32, SD = 1.50). In terms of education, there were differences in the mean score (*p* < 0.001), and the mean score increased as the education level increased.

.The mean A score was 3.81 (SD = 0.94). There were significant differences in the score according to age, marital status, labor force status, and Hukou (*p* < 0.05). The score was higher in males than in females. Monthly income did not significantly influence the A score. Hukou influenced the A score, as the A score was higher among participants living in urban areas than among those living in rural areas.

The mean P score was 3.02 (SD = 0.001). There were significant differences in the score according to sex, education level, marital status, monthly income, and residential area (*p* < 0.05). The score was higher in female participants than in male participants. Additionally, participants aged 35–44 years had the highest score. However, there were no significant differences in the P score according to age.

### 4.4. Assessment of the Overall Score

The mean total KAP score was 12.23 (SD = 2.23). The scores for male and female participants were similar. Participants with the highest education level usually had the highest mean total KAP score (13.10, SD = 1.956). The total scores did not show statistical differences with respect to sex, marital status, and occupation.

### 4.5. Correlation among Heat Wave-Related K, A, and P Scores 

Correlation analyses suggested a significant positive correlation between K and A scores (r = 0.068, *p* < 0.01), K and P scores (r = 0.239, *p* < 0.01), and A and P scores (r = 0.214, *p* < 0.01); however, the correlations were weak, so the sub-scales were not strongly correlated (Table 3).

### 4.6. Main and Interactive KAP Effects on Heat Illnesses 

In our survey, 436 (19.4%) participants answered that they experienced heat-related illnesses in the previous year, and of these participants, 195 reported that they experienced heatstroke. Additionally, 339 participants had symptoms of discomfort or self-medication because of heat and 98 participants had experienced both. 

Table 4 presents the odd ratios (ORs) that were calculated based on the four unconditional logistic regression analyses. After adjustments for potential confounding variables, the main effects analysis for K, A, and P showed that only the main effect of K was significant (*p* < 0.001). When the group of participants with K scores of >5 was set as the reference group, the group of participants with K scores of 3–5 was found to have a high risk of heat-related illnesses (OR = 1.43, 95% confidence interval CI: 1.14–1.78; adjusted odds ratio aOR = 1.53, 95% CI: 1.22–1.93). Statistical assessments performed on different groups of participants categorized according to the A and P scores found that the A and P scores were not significantly associated with the occurrence of heat-related illnesses. Stratified analyses were performed in order to assess the interactive effects among the K, A, and P scores. Participants with A and P scores of >3 had protective interactive effects with regard to the occurrence of heat-related illnesses (OR = 0.30, 95% CI: 0.13–0.67; aOR = 0.29, 95% CI: 0.13–0.64). Participants with an A score of <3 and a P score of ≥3 had the highest risk of heat illnesses (OR = 2.69, 95% CI: 1.27–5.68) during the heat-wave period.

### 4.7. Demographic Characteristics of Participants with a High Level of Practice but a Negative Attitude during Heat Waves

There were 124 participants with a high level of practice but a negative attitude to heat protection, and 49.2% of these participants were male. Among the participants, 66.9% had a low education level, 62.9% had a low income (less than 3000 RMB), and 46.8% were from urban areas (Table 5). 

### 4.8. Reliability and Construct Validity of the Questionnaire

The reliability and construct validity of the 13 questions in the KAP questionnaire on heat waves were assessed using reliability analysis and exploratory factor analysis, respectively. Cronbach’s α-coefficient of the reliability of the total scale was 0.770, which was considered acceptable for internal consistency reliability [22]. In the assessment of validity, the Kaiser–Meyer–Olkin measure determined a coefficient of 0.846, indicating that the samples were adequate and representative. The value in Bartlett’s test of sphericity was 5760 (*p* < 0.001), indicating that the correlation matrix was unlikely to be an identity matrix and was suitable for factor analysis [23].

## 5. Discussion

Studies have found that heat waves or extremely high temperatures have negative effects on human health [24,25]. However, few studies have explored the public’s KAP with regard to heat waves. Studies on heat wave-related KAP in China may be greatly significant as China is severely affected by heat events; hence, the findings of such studies may essentially transform or modify political, economic, and social actions so that the effects and risks from global or regional heat wave events can be addressed [12,26]. 

To our knowledge, this is the first study in East China to investigate the public’s KAP with regard to heat waves. The majority of the participants had a high KAP score. However, some subgroups showed low KAP scores because of demographic factors, environmental differences, and different social ties.

In this survey, most participants stated that they would protect themselves on days when the temperature was high. However, this finding is different from the results of a study performed in America [21] where only about half of the participants protected themselves when the temperature was high. Male participants, young participants, and participants living in rural areas had a low mean A score, although they are considered to be more vulnerable to the effects of heatstroke [12]. These findings are similar to those of previous studies [12,27] that reported a relatively lower risk perception to heat waves among vulnerable populations. These findings may be partially explained by two factors. First, the majority of outdoor employees were young men who may be more willing to take risks and believe that they can handle heat [21]. Second, rural populations usually have a low level of education and insufficient knowledge, and therefore, they are not aware of methods to protect themselves during high temperature days. Moreover, they do not consider themselves to be vulnerable to heat illnesses. There is an urgent need to take effective actions, such as implementation of education programs about heat waves, to increase awareness among such populations.

Knowledge is important to reduce the adverse impact of climate change. In the present study, all demographic characteristics of the participants were associated with the K score. Participants with high education levels had high mean K scores. Additionally, the K score was higher among young participants than among old participants, and this may be because young individuals can obtain information on heat waves through more channels. For example, young individuals may access information through educational programs on various media, such as television, radio, the internet, smart phones, and/or newspapers [28]; whereas, old individuals obtain information mostly through traditional media as they may face challenges and may have to put in more effort when using new technologies, such as the internet and smart phones. Media channels, such as television and radio, should be used to provide information during a heat-wave period in order to reduce the mortality rate among elderly individuals. Government offices should realize that providing educational programs through various methods is very important. Certain age groups do not appear to have adequate ways to access heat wave-related knowledge; moreover, the entire population does not appear to be aware of certain important information. In our survey, the public’s awareness of the causes of the greenhouse effect and rational prescription drug use was rather low, with 26% and 42.2% of the participants providing accurate responses to those questions, respectively. This may be because knowledge regarding these factors is not as common as knowledge regarding the role of green plants in cooling. Hence, people are simply unaware of the importance of such knowledge. Furthermore, local governments may have insufficient resources or may not have taken adequate efforts to raise public awareness by promoting such information. Knowledge may be related to the level of economic development, as high economic status is often associated with high education levels. This could explain why populations in developed countries tend to be aware of the current situation of the global environment [20,21,29]. Therefore, further efforts from individuals, local governments, and national institutions are needed to improve the KAP with regard to heat waves. 

The majority of participants took preventive measures during heat waves. However, a considerable number of participants did not take any measures. Additionally, only 56.1% of the participants drank water when they were thirsty, and 11.4% of the participants arranged outdoor activities on days with high temperatures. Sheridan et al. found that only half of the participants drank more water than usual during the warmest parts of the day [21], and this is consistent with our finding. Perspiration is required to lower body temperatures when the ambient temperature exceeds 34 °C. The best method to overcome excess loss of water is to drink sufficient water so that the perspiration cycle is appropriate. Many participants took personal measures only based on whether they felt thirsty owing to lack of knowledge on perspiration. It is important to determine the most appropriate intake of water, as Yong et al. found that people who had a history of excessive water intake had a high risk of hyponatremia during heat waves [30]. Fluid intake guided by thirst levels and urination is considered by experts to be appropriate for heat wave protection. Our study also found that the P score was higher in female participants than in male participants. Differences in risk awareness between male and female individuals may explain this difference. Considering the general family-related roles and domestic responsibilities of women, they are more likely to care about the safety of their family members and friends [28]; hence, they may pay more attention to the safety of their loved ones on days with extreme temperature or weather conditions. It has been shown that stressful events, such as heat-related events, have a greater effect in women than in men [31]. Therefore, women are more likely to notice changes in the surrounding environment directly by recognizing changes in their own emotional or psychological status or indirectly by warnings from others who recognize their emotionally or psychological changes, and therefore, they can take preventive actions in time. In the present study, the P score was higher in urban populations than in rural populations, and the impact of the urban heat island (UHI) effect cannot be ignored. The UHI effect is a well-documented phenomenon wherein a thermal anomaly exists in urban areas when compared with proximate rural landscapes [32,33,34]. It could cause higher temperatures in urban areas than in places that are less dense and have more vegetation cover, such as rural or suburban areas [35]. The UHI effect has increased in Jinan City from 1970 to 2012 [36]. Some studies have suggested that there is a significantly elevated risk of death on hot days in heat island areas [37,38,39]. Therefore, the urban population should not only take protective measures, but also take measures to mitigate the UHI effect.

Pearson correlation analysis indicated that there is limited correlations between K and A scores, and weak correlations between A and P scores, which is consistent with the results of the interactive effect analysis in this study. This finding of a correlation between the A and P scores was consistent with the results of a study by Liu et al. performed in Guangdong, China [12]. The authors reported that risk perception was positively correlated with adaptive behavior.

A series of multivariate logistic regression analyses were used to assess the main and interactive effects of KAP on heat-related illnesses. As explained earlier, a low K score may be associated with high health risks, and therefore, such individuals may experience severe heat-related illnesses. In addition, the results showed that the main effects of A and P had no significant differences with respect to heat illnesses, which contradicts the expected result. Generally, a positive attitude or perception to heat protection and preventive practice should reduce the adverse effects of high temperature [12,40]. In the questionnaire, there were few questions for assessing the A and P scores, which placed uneven weight on the total KAP score. The scores may not be sufficient and effective to identify a correlation of heat illnesses with the A or P score, as well as a relationship among the K, A, and P scores. Therefore, further studies with more questions for assessing the A and P scores are needed to explore their correlations with heat-related illnesses.

Stratified analysis was used to determine the interactive effects among the K, A, and P scores. Among the participants, those with high awareness and those who undertook adaptation practices had a low incidence of heat-related illnesses. Therefore, people who are aware of the importance of a preventive attitude and protective practice may have a low risk of heat-related illnesses. Another important finding is that participants with a high level of protective practice (≥3) but a negative attitude to heat protection (<3) during heat waves had the highest risk of heat illnesses, which is inconsistent with the widespread view that people with low protection awareness and limited adaptation behaviors are usually affected by heat illnesses. We further analyzed the demographic characteristics of this subgroup to determine the reasons for our finding (Table 5). Most participants in this subgroup had a low income, were from rural areas, and had low education levels. Additionally, most of the participants took actions; however, they had a high prevalence rate of heat illnesses. Therefore, some other factors may have been present. These individuals may not have been able to afford cooling equipment, such as air conditioners, with their low income. Moreover, they may not know how to adapt to heat waves, as few education resources are available in rural areas. Furthermore, owing to the limited transportation resources in rural areas and/or in areas with individuals having a low economic status, the individuals in these areas may not be able to access places, such as shopping malls and natatoriums, which are mainly located in urban areas, to avoid heat in summer. Therefore, more attention should be paid to vulnerable sub-populations, and special adaptation strategies should be designed for such populations. 

The present study has some limitations. First, this cross-sectional study only investigated regional KAP; thus, caution should be applied when generalizing the results to other cities or regions of the world. Second, because of the cross-sectional design of the study, a cause-effect relationship between KAP and heat-related illnesses could not be appropriately determined. To further explore the causal relationship between KAP and heat-related illnesses, we performed a quasi-experimental study on KAP in 2015, which is commonly used to measure behavior in communities before and after an intervention. Third, bias was not adequately addressed. For instance, although the interviewers were trained before the survey, there may have been some investigation bias during the interview process. Moreover, recall of previous experiences of heat illness may introduce bias. Furthermore, there may have been social desirability bias, as some participants may have provided answers that they believed were in the favor of the survey. Finally, the KAP approach cannot be used to explore perceptions and attitudes in depth. Despite these limitations, the findings of the present study are useful for policy implementation. In the future, relevant policy makers, such as emergency planners and health professionals, should pay close attention to the protection of the population, especially vulnerable groups. A series of measures can be implemented. First, a multi-level healthcare response network should be established to ensure that patients at the community level are identified and administered timely treatment. Second, the KAP level of community workers who provide special services to community residents should be enhanced. These workers can disseminate knowledge among residents through face-to-face communication. Finally, the government should use all available channels (newspapers, television, radio, the internet, telephone, etc.) to spread heat-related knowledge that will help prevent heat illnesses.

## 6. Conclusions

The KAP scores with regard to heat waves were relatively high among the participants from Licheng District. However, some participants did not consider themselves to be potentially vulnerable, and they had low KAP scores. The local government should focus on health education to improve awareness regarding the negative effects of heat waves among the entire population, especially vulnerable groups, such as elderly individuals, individuals with low education levels, those with low income levels, and those living in rural areas. There was a low rate of awareness regarding knowledge and practices. Thus, there is an urgent need to ensure the dissemination of factual knowledge and actions that can be undertaken among the entire population. The results indicate that low knowledge score were associated with a high risk of heat-related illnesses. Individuals who were aware of the importance of both preventive attitudes and protective practices had a low risk of heat-related illnesses. Therefore, governments should further develop risk-awareness strategies that increase awareness and knowledge regarding the adverse health impact of heat and help in planning measures to improve the ability of individuals to cope with heat waves.

## Figures and Tables

**Table 1:** Demographic characteristics of the 2241 survey participants.

Characteristic	Category	n	Proportion (%)
Sex	Male	1021	45.6
	Female	1220	54.4
Age (years)	15–24	263	11.7
	25–34	564	25.2
	35–44	399	17.8
	45–54	436	19.5
	55–64	276	12.3
	≥65	303	13.5
Education level	No formal education	100	4.5
	Elementary school	200	8.9
	Junior middle school	543	24.2
	Senior middle school	667	29.8
	Bachelor level	654	29.2
	Master level or above	71	3.2
	Unanswered	6	0.2
Marital status	Unmarried	363	16.2
	Married	1764	78.7
	Divorced	13	0.6
	Widowed	89	4.0
	Unanswered	12	0.5
Labor force status	Employed *	1505	67.2
	Unemployed	230	10.3
	Retired	322	14.4
	Student	184	8.2
Monthly income (RMB)	<2000	562	25.1
	2000–3000	758	33.8
	3000–5000	616	27.5
	5000–10,000	225	10.0
	>10,000	40	1.8
	Unanswered	40	1.8
Hukou	Urban	1379	61.5
	Rural	862	38.5

* Including agricultural worker, police officer, teacher, office worker, doctor, service worker, and other types of workers. RMB: Renminbi (Chinese currency; US$1 = RMB 6.119 in 2014).

**Table 2:** Responses to heat wave-related knowledge, attitude, and practice items.

Items	Question	Category	n (%)
Knowledge	Can sprinklers in open grounds and fans play a role in cooling?	Yes	1845 (82.3)
		No	378 (16.9)
	If you wear dark clothes, will you feel cool in summer?	Yes	603 (26.9)
		No	1614 (72.1)
	Should windows and doors be opened at noon on hot days?	Yes	859 (38.3)
		No	1357 (60.6)
	Are fever, fatigue, and chest tightness common symptoms of heat stroke?	Yes	1858 (82.9)
		No	363 (16.2)
	Can some medicines increase the risk of heatstroke?	Yes	946 (42.2)
		No	1252 (55.9)
	Can death be caused by high temperature?	Yes	1891 (84.4)
		No	332 (14.8)
	Is the greenhouse effect mainly caused by the depletion of the ozone layer?	Yes	1617 (72.2)
		No	582 (26)
	Can green plants play a role in cooling?	Yes	1877 (83.8)
		No	350 (15.6)
Attitude	Do you intend to take sunstroke prevention measures if a temperature warning is released?	Very much	570 (25.4)
		Much	926 (41.3)
		Some	524 (23.4)
		So so	201 (9.0)
		Not at all	20 (0.9)
Practice	Do you drink water only when you are thirsty?	Yes	1258 (56.1)
		No	983 (43.9)
	Do you try to arrange outdoor activities at cooler times?	Yes	1986 (88.6)
		No	255 (11.4)
	When you go out, do you implement good sunstroke prevention measures?	Yes	1793 (80)
		No	448 (20)
	Do you pay more attention to the elderly, children, or weaker family members?	Yes	2021 (90.2)
		No	218 (9.8)

**Table 3:** Correlations among knowledge, attitude, and practice scores.

Variable	Knowledge Score	Attitude Score	Practice Score
Knowledge score	1		
Attitude score	0.068 *	1	
Practice score	0.239 *	0.214 *	1

* *p* < 0.01.

**Table 4:** Main and interactive effects of knowledge, attitude, and practice on heat illnesses in Licheng District.

Variable	Category	Heat Lllness in the Current Year					
		Without Heat Illness n (%)	With Heat Illness n (%)	Model I OR (95% CI)	Model II aOR (95% CI)	Model III OR (95% CI)	Model IV aOR (95% CI)
Knowledge	<3	69 (80.2)	17 (19.8)	1	1		
	3–5 ^a^	711 (77.7)	204 (22.3)	1.17 (0.68–2.02)	1.14 (0.66–1.98)		
	>5	904 (83.4)	180 (16.6)	0.81 (0.47–1.41)	0.74 (0.42–1.29)		
Attitude	<3	167 (79.1)	44 (20.9)	1	1		
	≥3	1562 (80.8)	371 (19.2)	0.92 (0.64–1.32)	0.92 (0.64–1.32)		
Practice	<3	358 (79.6)	92 (20.4)	1	1		
	≥3	1370 (80.9)	323 (19.1)	0.97 (0.74–1.27)	0.98 (0.74–1.28)		
Knowledge × Attitude	<3 × <3	2 (9.5)	19 (90.5)			1	1
	3–5 × <3	24 (24.2)	75 (75.8)			3.04 (0.66–14.01)	2.30 (0.48–10.89)
	>5 × <3	18 (19.8)	73 (80.2)			2.34 (0.50–10.99)	1.75 (0.36–8.44)
	<3 × ≥3	16 (23.2)	53 (76.8)			2.87 (0.60–13.65)	2.17 (0.44–10.65)
	3-5 × ≥3	190 (22.1)	668 (77.9)			0.31 (0.06–1.59)	0.43 (0.08–2.26)
	>5 × ≥3	165 (16.4)	841 (83.6)			0.27 (0.05–1.44)	0.36 (0.06–1.93)
Knowledge × Practice	<3 × <3	11 (23.9)	35 (76.1)			1	1
	3-5 × <3	54 (23.1)	180 (76.9)			0.95 (0.45–2.01)	0.87 (0.41–1.86)
	>5 × <3	27 (15.8)	144 (84.2)			0.59 (0.27–1.32)	0.52 (0.23–1.16)
	<3 × ≥3	7 (15.9)	37 (84.1)			0.60 (0.21–1.73)	0.54 (0.18–1.59)
	3–5 × ≥3	160 (22.1)	563 (77.9)			1.57 (0.52–4.78)	1.77 (0.57–5.49)
	>5 × ≥3	156 (16.8)	770 (83.2)			1.79 (0.57–5.64)	1.97 (0.61–6.28)
Attitude × Practice	<3 × <3	11 (12.2)	79 (87.8)			1	1
	<3 × ≥3	81 (22.4)	280 (77.6)			2.59 * (1.22–5.53)	2.69 * (1.27–5.68)
	≥3 × <3	33 (27.3)	88 (72.7)			2.01 * (1.01–3.98)	2.08 * (1.05–4.09)
	≥3 × ≥3	290 (18.4)	1281 (81.6)			0.30 * (0.13–0.67)	0.29 * (0.13–0.64)
*p*-value for main effect of knowledge				0.006	0.001		
*p*-value for the interaction of attitude ≥3 × practice ≥3						0.003	0.002

* *p* < 0.05; OR: odds ratio; aOR: adjusted odds ratio; CI: confidence interval; ^a^ knowledge >5 as the reference, OR = 1.433, 95% CI: 1.14–1.78; aOR = 1.539, 95% CI: 1.22–1.93; confounding variables: sex, age, education, marital status, labor force status, monthly income, and Hukou.

**Table 5:** Demographic characteristics of participants with a high level of practice but a negative attitude during heat waves (*n* = 124).

Characteristic	Category	n	Proportion (%)
Sex	Male	61	49.2
	Female	63	50.8
Age (years)	15–34	56	45.2
	35–54	44	35.5
	55–74	20	16.1
	75–86	4	3.2
Education level	Senior middle school or lower	83	66.9
	Higher education	41	33.1
Marital status	Unmarried	36	29
	Married	24	19.4
	Divorced	96	77.4
	Widowed	4	3.2
Labor force status	Employed	87	70.2
	Unemployed	12	9.7
	Retired	15	12.1
	Student	10	8.1
Monthly income (RMB)	<2000	36	29
	2000–3000	42	33.9
	3000–5000	28	22.6
	5000–10,000	14	11.3
	>10,000	4	3.2
Hukou	Urban	66	53.2
	Rural	58	46.8

## Abbreviations

KAP	Knowledge, attitude, and practice
SD	Standard deviation
OR	Odds ratio
aOR	Adjusted odds ratio
CI	Confidence interval
